# Value of DWI- ADC and FUSION T2-DWI in the management of oncological patients

**DOI:** 10.1186/1470-7330-15-S1-P52

**Published:** 2015-10-02

**Authors:** M Nazar, F Saguier, L Alarcon, M Pascuzzi, M Wirtz, E Eyheremendy

**Affiliations:** 1 Hospital Aleman, Buenos Aires, Argentina

## Learning objectives

Describe DWI MRI and fusion T2-DWI techniques and findings for detection and characterisation of tumours, list the current and potential applications of DWI in cancer patient management and review causes of false-positive and false-negative results in lesion detection.

## Content organisation

The detection and characterisation of malignant lesions can often be difficult, principally when the disease is small or when the tumour is combined with normal tissues.

Forty five patients with malignancies underwent both DWI MRI and T2-DWI to detect and characterise primary and metastatic tumours. We acquired DWI (b0, b400, b600), FAT SAT T1w imaging before and after gadolinium administration and STIR images. The standard reference was histopathology findings.

## Conclusion

DWI-MRI and fusion T2-DWI imaging is a powerful clinical tool for directing the care of patients with cancer. Three- dimensional fusion imaging of high *b* value DW-MRI with anatomic imaging have a number of utilities including data presentations to clinicians for detecting and guiding biopsy to variable tumour.

